# Case report: the effects of cerebellar tDCS in bilingual post-stroke aphasia

**DOI:** 10.3389/fnhum.2023.1173178

**Published:** 2023-07-20

**Authors:** Silke Coemans, Esli Struys, Kyrana Tsapkini, Philippe Paquier, Dorien Vandenborre, Stefanie Keulen

**Affiliations:** ^1^Brussels Centre for Language Studies (BCLS), Vrije Universiteit Brussels, Brussels, Belgium; ^2^Center for Neurosciences (C4N), Vrije Universiteit Brussels, Brussels, Belgium; ^3^Department of Neurology, Johns Hopkins School of Medicine, Baltimore, MD, United States; ^4^Department of Cognitive Science, Johns Hopkins University, Baltimore, MD, United States; ^5^Center for Research in Cognition and Neurosciences (CRCN), Université Libre de Bruxelles, Brussels, Belgium; ^6^Department of Translational Neurosciences (TNW), Universiteit Antwerpen (UA), Antwerp, Belgium; ^7^Health and Wellbeing Research Unit, Thomas More University of Applied Sciences, Antwerp, Belgium

**Keywords:** cerebellum, transcranial direct current simulation, bilingualism, aphasia, neurolinguistics, executive functions (EFs), bilingual aphasia

## Abstract

Transcranial Direct Current Stimulation may be a useful neuromodulation tool for enhancing the effects of speech and language therapy in people with aphasia, but research so far has focused on monolinguals. We present the effects of 9 sessions of anodal cerebellar tDCS (ctDCS) coupled with language therapy in a bilingual patient with chronic post-stroke aphasia caused by left frontal ischemia, in a double-blind, sham-controlled within-subject design. Language therapy was provided in his second language (L2). Both sham and anodal treatment improved trained picture naming in the treated language (L2), while anodal ctDCS in addition improved picture naming of untrained items in L2 and his first language, L1. Picture description improved in L2 and L1 after anodal ctDCS, but not after sham.

## Introduction

More than half of the world’s population is bilingual: in a survey conducted by the European Commission in 2012, just over half (54%) of the European respondents reported being functionally bilingual (European Commission, 2012). This number is only expected to rise in our age of globalization, and with it, the incidence of bilingual aphasia, caused by for instance stroke or neurodegenerative disease (Ansaldo and Saidi, 2014). Aphasia is a neurological language disorder affecting language production and comprehension to different degrees, resulting in a specific aphasia subtype, classified within the spectrum of fluent (receptive) or non-fluent (expressive) aphasias. Bilingual aphasia comes with different implications for diagnosis and rehabilitation as compared to monolingual aphasia. For instance, patterns of recovery can vary in bilinguals with aphasia (Paradis, 2001). Parallel, or simultaneous, recovery is the most common type of recovery: both languages are restored simultaneously and to equal extent. In differential recovery, the languages do not recover in equal patterns, while in selective recovery one language does not recover at all (Fabbro, 2001). Several factors can influence these different patterns. A recent meta-analysis found that bilingual speakers with aphasia generally perform better in their first language (L1) than in their second language (L2), and that the magnitude of this effect is moderated mainly by age of acquisition (with 7 years of age being the cut-off point between “early” and “late” acquisition) and to a smaller extent by premorbid language proficiency and frequency of use (Kuzmina et al., 2019). These different patterns of recovery and the factors that might influence them, are important (and interesting) to consider when assessing bilingual aphasia. Another distinction between bilingual and monolingual aphasia, is that treatment comes with different challenges, e.g., the unresolved question of whether to focus on a single language (and which?) or both languages (if even possible) (Faroqi-Shah et al., 2010). Further, bilingual patients with aphasia may exhibit pathological code-switching or code-mixing (Fabbro et al., 2000), thought to be caused by an impairment of “bilingual language control” (BLC). BLC refers to the ability to avoid interference of the two languages, and to select one language over the other depending on the communicative context (Calabria et al., 2018). Neurobiologically, bilinguals are thought to differ from monolinguals in the sense that their linguistic functions are effectuated by similar neural circuits as in monolinguals, but with an increased demand on executive and attention control processes (Abutalebi, 2008). Regarding these differences between bi-and monolinguals, research needs to look at how aphasia treatment protocols can be made appropriate for bilinguals with aphasia.

Neuromodulation with transcranial direct current stimulation (tDCS) has been demonstrated to be useful for increasing the efficacy of speech and language therapy in individuals with aphasia [for a recent review see Marangolo (2020)]. To our knowledge, no research has been published regarding the use of (cerebellar) tDCS in bilinguals with aphasia. In general, methodological questions about the use of tDCS in aphasia remain, one of them being which area(s) of the brain are most suitable to be stimulated in combination with the language therapy (Marangolo, 2020). Neuroimaging and anatomical studies have revealed crossed anatomical connections between the lateral cerebellar hemispheres and frontal and parietal association areas in the contralateral cerebral cortex, and indicate that the right cerebellum plays a role in language functions and processes of executive and attention control (Schmahmann, 1991, 2001; Middleton and Strick, 2001; Murdoch, 2010; Stoodley, 2012; Mariën et al., 2014). As these latter processes are known to be important for language control in bilinguals [e.g., Abutalebi (2008), Filippi et al. (2020)], this makes the cerebellum a strong candidate location for stimulation in individuals with bilingual aphasia. Further, studies have found increased cerebellar volume in bilinguals, compared to monolinguals [e.g., Pliatsikas et al. (2014)]. Lesion studies have found that damage to the right cerebellum is associated with deficits in a variety of language tasks (Marien et al., 1996, 2000; Baillieux et al., 2010). In healthy adults, tDCS stimulation of the right cerebellum improved verbal fluency (Turkeltaub et al., 2016) and verb generation (Pope and Miall, 2012). With regard to monolingual post-stroke aphasia, cerebellar tDCS (ctDCS) has been shown to improve spelling to dictation (Sebastian et al., 2016), verb generation (Marangolo et al., 2018) and picture naming (Sebastian et al., 2020). Finally, stimulating the spared cerebellum, a modulator of several cognitive and language functions, could possibly be applied to a variety of patients with different sites of cortical lesions.

We aimed to evaluate (1) whether ctDCS stimulation applied to the right posterolateral cerebellum has a positive impact on language outcomes in the treated language (language of therapy) and/or non-treated language, (2) which linguistic functions benefit from ctDCS, and (3) whether the executive control circuits benefit from ctDCS application and associated language therapy in stroke, via an attention network test (ANT).

## Methods

### Participant

**FIGURE 1:**
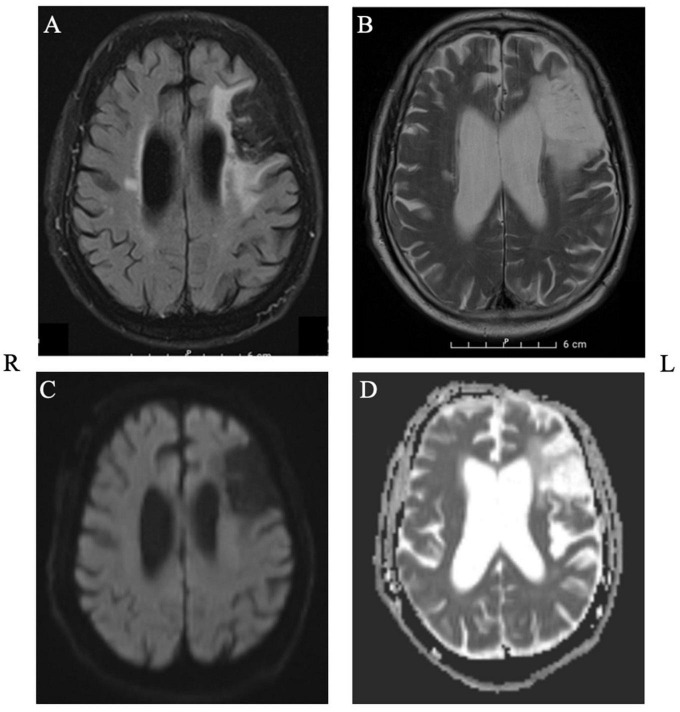
Structural MRI depicting the extent of the lesion in axial plane. Panel **(A)**: T2_FLAIR, Panel **(B)**: T2_TSE, Panel **(C)**: DWI, Panel **(D)**: ADC.

Mr. J was a 72-year-old right-handed man who suffered from left frontal ischemia (inferior and middle frontal gyrus, until pars opercularis), consistent with an infarct of the left middle cerebral artery (MCA), 3 years prior to our current study. He presented to the emergencies with expressive aphasia and a paresis of the facial nerve. The MRI revealed a major peripheral chronic left frontal media infarction and a chronic cortico-subcortical infarction at the level of the precentral gyrus on the right (Figure 1). Aside from aphasia, the neurological examination at the time of intervention indicated normal awareness, normal force distribution, intact sensitivity and good coordination and reflexes.

Regarding his language history, he learnt French and Dutch from birth, but during his childhood the language most often used at home and at school was French, which Mr. J identified as his first language (L1). He grew proficient in Dutch, his second language (L2), after being immersed in a Dutch school environment from the age of 11. As an adult, he used both languages daily to a similar extent, in his private as well as his professional life. As such, he reported to be highly proficient in French and Dutch pre-morbidly, with French being his (slightly) better language [self-reported rate of 10/10 for French, and 9/10 for Dutch, in all language modalities, LEAP-Q (Marian et al., 2007)]. After his stroke, during his hospitalization and onward, he received speech-and language therapy. Therapy was provided in Dutch, his L2, as the city he lives in is Dutch speaking, and where care is generally provided in Dutch. 9 months prior to inclusion in our study, his last language testing in L2 was indicative of non-fluent aphasia, with poor results on tests for word fluency, oral naming, oral and written sentence comprehension, and oral and written picture description. Spontaneous speech was very laborious and telegraphic in style, with large word finding difficulties. There had been no testing of his L1 prior to the start of our research.

### Behavioral and neurolinguistic assessment

We conducted the Mini-Mental State Examination (Folstein et al., 1975), a screening tool for global cognitive functioning, and the LEAP-Q questionnaire (Marian et al., 2007) to collect self-reported L1 and L2 proficiency and experience data. Further, we established a baseline assessment of language abilities, by use of subtests of the Bilingual Aphasia Test [BAT, Paradis (2011)], Boston Naming Test [BNT, L1, and L2, Kaplan et al. (1983)], Picture Description tasks in L1 and L2 [from the Comprehensive Aphasia Test-NL (Visch-Brink et al., 2014) and Boston Diagnostic Aphasia Examination, Cookie Theft Picture (Goodglass, 1972)], and a personalized naming test, with different items for both tDCS stimulation phases (27 different but matched items for each stimulation phase), that were matched to items of the untrained BNT with regards to number of syllables, word frequency, and imaginability. Further, we assessed executive functions, by use of the Attention Network Test (ANT) (Fan et al., 2002). The ANT combines Posner’s cuing task (Posner, 1980) with Eriksen’s flanker task (Eriksen and Eriksen, 1974) and is a test of attentional networks (the alerting, orienting, and executive network), of which we looked into the executive network. The measure of executive network efficiency in the ANT, the “Flanker effect,” is based on response times to stimuli in different conditions of congruency. The incongruent condition presents conflicting information, requiring inhibition to elicit a correct response, leading to longer reaction times than in the congruent condition. Scores of the congruent condition are subtracted from scores in the incongruent condition, and is referred to as the “Flanker effect,” with a lower Flanker effect indicating greater executive network efficiency. See Table 1 for baseline test scores. The participant was assessed before both stimulation type treatment phases [T1 (sham) and T3 (tDCS)] for baseline scores, immediately after 3 weeks of the treatment [T2 (sham) and T4 (tDCS)] to evaluate changes in scores after therapy, and at 8 weeks follow-up evaluation [T3 (sham) and T5 (tDCS)].

**TABLE 1:** Patient characteristics and baseline performances in French (L1) and Dutch (L2).

Patient characteristics		
Patient age (years: months)	72:11	
Time post-stroke (years: months)	3:2	
Years of education	15	
Language background*	**L1**	**L2**
Age of acquisition	Birth	11
Proficiency pre-stroke (all modalities, 10)	10	9
Percentage exposure pre-stroke (100)	50	50
Percentage exposure post-stroke (100)	70	30
Mini-Mental State Examination (30)	26	
Picture naming**	**L2**	**L1**
Trained items (81)	N/A	25
Untrained items (Boston Naming Test) (177)	132	105
**Bilingual Aphasia Test**	**L2**	**L1**
**Part B**		
Verbal auditory discrimination (18)	18	8
Syntactic comprehension (66)	59	46
Repetition of words and nonsense words (30)	27	23
Repetition of sentences (7)	7	6
Verbal fluency–semantic	6	6
Verbal fluency–phonetic	1	1
Semantic opposites (10)	10	9
Reading out loud–words (10)	10	10
Reading out loud–sentences (10)	10	10
Copying (5)	5	5
Dictation (5)	5	5
Reading comprehension–words (10)	10	10
Reading comprehension–sentences (10)	10	10
Naming (6)	6	6
**Part C**		
Grammaticality judgements (8)	7	2
	L1 into L2	L2 into L1
Word recognition (5)	4	4
Word translation (10)	7	8
Sentence translation (18)	10	10
Picture Description***	**L2**	**L1**
# Words	74,0	26
# Nouns	16,0	7
# Different Nouns	11,0	6,5
# Utterances	14,5	6,5
MLU	4,0	3,6
% correct utterances	48	22
Phonemic Paraphasia	0,0	0,0
Semantic Paraphasia	0,5	0,5
Code Switching	0,0	0,0
Code Mixing	0,0	0,0

*Maximum test scores (not total number of picture naming items) are listed within brackets. Trained picture naming consisted of 27 picture items per phase (sham/anodal ctDCS), with different items for each phase. The untrained Boston Naming Test consists of 59 picture items. **Averaged scores of the picture description tasks from (1) Comprehensive Aphasia Test-NL (Visch-Brink et al., 2014) and (2) Boston Diagnostic Aphasia Examination, Cookie Theft Picture (Goodglass, 1972).

### Intervention: tDCS

Cerebellar tDCS (ctDCS) of 2 mA was delivered for 20 min during speech and language therapy, using a direct current stimulator (Oasis Pro, Mind Alive Inc., Canada), via a 3 cm X 3 cm saline-soaked sponge anode placed over the right posterolateral cerebellum (4 cm lateral to the inion and 1 cm down, over right lobule VII), with the reference electrode on the right deltoid muscle (Sebastian et al., 2020). In our randomized within-subject controlled design, the patient received both sham (no stimulation) and anodal ctDCS combined with online language therapy, three sessions per week for 3 weeks, for a total of nine sessions per stimulus condition (sham/anodal ctDCS). Language therapy lasted for another 10–15 min after removal of the tDCS electrodes. Between the two stimulus conditions, a 2-month break was introduced, to avoid possible interference of the aftereffects of ctDCS stimulation. The patient, evaluator and therapist were blinded to the stimulus condition. During the 2-month break, the patient received speech and language therapy in the same manner as before entering our study: he had a prescription for 30 min per week, in which focus mainly lay on word-finding, using a variety of exercises, such as word fluency exercises. The speech and language therapist did not train picture naming specifically prior to our study or during the 2-month break.

### Intervention: language treatment

Mr. J received language treatment in L2, mainly focusing on his problems with word-retrieval, including a picture naming treatment. A list of therapy items (trained items) was compiled, matched with the untrained items of the BNT (consisting of 59 items) (Kaplan et al., 1983) with regard to word frequency, syllable length and imageability. From this list, pictures that could not be named correctly by the patient during at least one of two picture naming assessments, were included for therapy. The picture items chosen for therapy were different for each stimulation condition, with items for both conditions again matched with the BNT and with each other. This led to inclusion of 27 picture items, different for each stimulation condition (sham/anodal ctDCS). Picture items were scored in a similar manner as the BNT, where each named item can receive zero, one, two or three points, based on correctness, timing, presence of paraphasias etc. As such, the maximum score to be obtained with the personalized naming test, is a score of 81 (27 items times 3). The therapy procedure was as follows: Mr. J was shown a picture to name in L2, and if he could not name it within the first 10 s of seeing the picture, he was asked to describe features of it, as in semantic-feature analysis (e.g., Where do you find it? What does it do? Describe what it looks like?) (Boyle and Coelho, 1995). If he was still not able to name the item, he was provided with semantic cues, and additionally phonological cues to help facilitate naming. If naming was still not possible; the word was said out loud and the patient was asked to repeat it. Each therapy session was supplemented with other word-finding exercises, such as word fluency training (e.g., Please name 10 parts of the human body?).

### Statistical analysis

For each stimulation type (sham and tDCS), we compared scores pre-treatment with post-treatment, and pre-treatment with follow-up, with the baseline measurement time point of the second phase being the follow-up measure time point of the first phase. This means that in the sham phase, we compare T1 (baseline sham) with T2 (post-treatment), and T1 (baseline sham) with T3 (follow-up sham), and in the tDCS phase, we compare T3 (baseline tDCS) with T4 (post-treatment tDCS) and T3 with T5 (follow-up tDCS). For naming scores, we used McNemars test for correlated responses (McNemar, 1947).

The Flanker effect (difference incongruent and congruent condition ANT) was analyzed by means of ANOVA for repeated measures with “congruency type” (congruent and incongruent), “stimulation type” (sham and anodal) and “timing” (pre-treatment, post-treatment and follow-up) as the within-subject factors. A *post hoc* correction according to Bonferroni was applied if necessary.

## Results

### Baseline performance

The patient had an MMSE score of 26/30. On the Bilingual Aphasia Test (BAT), the patient scored better in his L1 compared to L2 on the subtests of verbal auditory discrimination (18/18, L1 vs. 8/18, L2), syntactic comprehension (59/66, L1 vs. 46/66, L2) and repetition of words and sentences (27/30, L1 vs. 23/30, L2). Semantic and phonetic fluency tests scores were identical for both languages, with a lower score for phonetic (1) than semantic (6) fluency. He obtained maximum scores in both languages on the other BAT subtests. On Part C, which comprises language pair-specific tests, his scores were the same for sentence translation (10/18) and word recognition tests (4/5) in both directions (from L1 into L2 and from L2 into L1), and similar for word translation (7/10 from L1 into L2, 8/10 L2 tino L1). On the grammaticality judgments test he scored better in L1 (7/8) than in L2 (2/8). In addition to the BAT, baseline testing (T1) consisted out of a personalized picture naming task in L2 (25/81), BNT in L1 (132/177) and L2 (105/177), picture description tests in L1 and L2, and the Attention Network Test (ANT). In the ANT congruent condition, response time was 1329.3 ms and accuracy was 89.2%. In the incongruent condition, response time was 1387.9 ms and accuracy was 78.7%.

### Effects of cerebellar tDCS on task performance

BAT syntactic comprehension scores in L1 improved significantly after sham (*p* < 0.05, χ^2^ = 7), and anodal cerebellar tDCS (cTDCS, *p* < 0.05, χ^2^ = 6) (see Table 2). Note: For the BAT we only present results with significant changes (see Supplementary Table 1 for all scores).

**TABLE 2:** Raw scores for trained words (L2), untrained words (L1 and L2), and picture description tasks (L1 and L2) at all assessment time points.

	Sham		ctDCS		
**Task**	**Pre-treatment T1**	**Post-treatment** **T2**	**Pre-treatment** **T3**	**Post-treatment** **T4**	**Two months post-treatment** **T5**
Picture naming*					
Trained items–L2 (81)	25	51 (***p* < 0.05)**	39**	68 (***p* < 0.05)**	54 (***p* < 0.05)**
Untrained (BNT)–L2 (177)	105	114 (*p* > 0.05)	123 (***p* < 0.05)**	142 (***p* < 0.05)**	137 (***p* < 0.05)**
Untrained (BNT)–L1 (177)	132	129 (*p* > 0.05)	143 (***p* < 0.05)**	152 (***p* < 0.05)**	149 (*p* > 0.05)
BAT–L1***					
Syntactic comprehension (66)	59	66 (***p* < 0.05)**	60 (*p* > 0.05)	66 (***p* < 0.05)**	60 (*p* > 0.05)
Repetition of words and nonsense words (30)	27	27 (*p* > 0.05)	25 (*p* > 0.05)	30 (***p* < 0.05)**	27 (*p* > 0.05)
Picture description L2****					
Number of Words	26	27,0	24,0	40,5	31,0
Number of Utterances	6,5	5,5	5,5	7,0	6,0
MLU	3,6	3,8	4,0	4,6	4,2
% correct utterances	22	40	57	75	61
Phonemic Paraphasia	0,0	0,0	0,0	0,0	0,0
Semantic Paraphasia	0,5	0,5	1,0	1,0	0,0
Code Switching	0,0	0,0	0,0	0,0	0,0
Code Mixing	0,0	0,0	1,0	0,0	0,0
Picture description L1****					
Number of Words	74,0	50,5	32,0	53,0	54,0
Number of Utterances	14,5	7,5	6,0	9,5	8,0
MLU	4,0	5,6	4,7	4,9	4,9
% correct utterances	48	59	49	58	52
Phonemic Paraphasia	0,0	0,0	0,0	0,0	0,0
Semantic Paraphasia	0,5	0,0	1,5	0,5	0,0
Code Switching	0,0	0,0	0,0	0,0	0,0
Code Mixing	0,0	2,0	1,0	0,0	0,0
Attention network test*****					
RT congruent trials	1329.323	1209.641 (vs. T1, *p* = 0.054)	1262.374 (vs. T1 *p* = 0.129, vs. T2, *p* = 1.000)	1069.016 (vs. T3, ***p* < 0.001**)	1094.532 (vs. T3 and T4**, *p* < 0.001**)
RT incongruent trials	1387.934	1381.161 (vs. T1, *p* = 1.000)	1372.39 1 1 (vs. T1 and T2, *p* = 1.000)	1368.77 (vs. T3, *p* = 1.000)	1381.582 (vs. T3 and T4, *p* = 1.000)

*Maximum test scores (not total number of picture naming items) are listed within brackets. T2 and T3 are compared to T1, with T2 immediately post-treatment and T3 2 months post-treatment for sham phase. T4 and T5 are compared to T3, with T4 immediately post-treatment and T5 2 months post-treatment for anodal ctDCS phase. McNemar’s test results (two-tailed, *p*-value) comparing the correct responses between pre-treatment and post-treatment, on each stimulus type (trained words L2, untrained words L2 and L1) are shown in italics. Bold indicates significant change. Trained picture naming consisted of 27 picture items per phase (sham/anodal ctDCS), with different items for each phase. The untrained Boston Naming Test consists of 59 picture items. **As picture naming items differed in each phase, McNemar’s test for paired items was not possible at T3. ***For the BAT we only present results with significant changes (see Supplementary Table 1 for all scores). ****Averaged scores picture description tasks (1) Comprehensive Aphasia Test-NL (Visch-Brink et al., 2014) and (2) Boston Diagnostic Aphasia Examination, Cookie Theft Picture (Goodglass, 1972). *****T2 and T3 are compared to T1, with T2 immediately post-treatment and T3 2 months post-treatment for sham phase. T4 and T5 are compared to T3, with T4 immediately post-treatment and T5 2 months post-treatment for anodal ctDCS phase. Repeated measures ANOVA comparing response times and accuracy between pre-treatment and post-treatment. Bold indicates significant change.

Scores for the trained personalized picture naming task improved significantly after sham (*p* < 0.05, χ^2^ = 22.09) and after anodal ctDCS (*p* < 0.05, χ^2^ = 13), albeit slightly more after anodal ctDCS. Improvement lasted significantly only after anodal ctDCS (*p* < 0.05, χ^2^ = 11.09). Untrained picture naming BNT scores in L2 (trained language) improved significantly only after anodal ctDCS, (*p* < 0.05, χ^2^ = 16.2), which lasted until follow-up (*p* < 0.05, χ^2^ = 15.3). Untrained picture naming BNT scores in L1 (untrained language) improved significantly after anodal ctDCS (*p* < 0.05, χ^2^ = 6), which did not last until follow-up. Word repetition scores in L1 remained the same after sham, but improved significantly after anodal ctDCS (*p* < 0.05, χ^2^ = 5).

Picture description test scores in his trained language (L2) improved after anodal ctDCS, but not after sham. Picture description test scores in his untrained language (L1) declined after sham but improved after anodal ctDCS.

With regard to analysis of response times of the ANT, a repeated measured ANOVA revealed a significant congruency type x stimulation type x timing interaction [*F*(2,99) = 12.4, *p* < 0.001, η^2^_p_ = 0.184]. After Bonferroni’s correction, a significant effect was found only during the tDCS stimulation condition, on the congruent condition of the ANT, with a significant decrease in RT between T3 (pre-treatment, RT 1262.374 ms) and T4 (post-treatment, RT 1069.016 ms) with *p* < 0.001 and between T3 (pre-treatment, RT 1262.374 ms) and T5 (follow-up, RT 1094.532 ms), with *p* < 0.001, while no significant difference emerged for the sham condition. A flanker effect (significant difference between RT on incongruent and congruent ANT trials) was present in all trials except for T1 (baseline sham measurement).

To summarize, trained L2 scores and syntax comprehension in L1 improved after sham and anodal ctDCS, while repetition scores, untrained naming in L1 and L2, picture description in L1 and L2, and RT scores on the congruent condition of the ANT improved after anodal ctDCS only.

## Discussion

We illustrated the case of an individual with chronic bilingual post-stroke aphasia, and our preliminary results indicated that cerebellar tDCS (ctDCS) may be a valuable tool to enhance the effects of speech and language therapy in bilingual patients. Our patient received nine sessions of speech and language therapy over 3 weeks, combined with sham and anodal ctDCS in a within-subject controlled design. Specifically, the patient’s trained syntax comprehension in L1 and picture naming scores in L2 improved significantly after sham and anodal ctDCS, with the latter improvements lasting until 2-month follow up only after anodal ctDCS. Importantly, his repetition scores in L1, untrained naming in L1 and L2, and picture description in L1 and L2 improved after anodal ctDCS only. To our knowledge, no other studies on the use of tDCS in bilingual aphasia have been published to date.

This case study offers five main findings. First, picture naming treatment with semantic feature analysis was efficient for this patient, with longer lasting effects after anodal ctDCS.

Second, anodal ctDCS lead to within-level generalization, with improvements in picture naming scores for untreated items. These two findings are in accordance with prior literature on the use of tDCS in post-stroke aphasia, where in general, tDCS is suggested to favor a generalized, long-term improvement of different language abilities under scrutiny (Zettin et al., 2021; Ding et al., 2022; Harvey and Hamilton, 2022).

Third, anodal ctDCS but not sham stimulation led to between-level generalization: we found significant improvements on the untrained repetition (L1) and picture description tasks (L1 and L2). Our findings of generalization of effects to untrained tasks in the tDCS phase, further adds to existing preliminary evidence of between-level generalization in tDCS research in post-stroke aphasia [e.g., Biou et al. (2019)] as well as primary progressive aphasia (Cotelli et al., 2014; Gervits et al., 2016; Roncero et al., 2017). Recent studies suggest that this generalization may be specific to the computation of the area of stimulation (Wang et al., 2022), and may be predicted by baseline structural (Zhao et al., 2020) and functional connectivity (Ficek et al., 2018; Tao et al., 2021; Wang et al., 2023) of stimulated areas with other areas involved in the language tasks. This may apply to our results: the stimulated cerebellum is known to contribute to the computation of (verbal) working memory, which is critical for performance on the auditory word repetition task (Desmond et al., 1997; Chen and Desmond, 2005; Nozari et al., 2010). Improved picture description task performance may be facilitated by improved word-finding abilities, and/or other aspects of language that may be supported by the cerebellum and its structural and functional connections to language-related areas in the cortex. More research is needed to elucidate the role the cerebellum plays in these other aspects of language. The improvements on the patient’s picture naming and picture description abilities in his trained language (L2) are very promising, as rehabilitation in this language had been stagnant for a long time. At baseline testing upon commencing our study, it appeared that his L1 had recovered more than L2, indicating a differential recovery pattern (Paradis, 2001). Many factors can influence the recovery patterns in bilingual aphasia, and for this patient, we can hypothesize that two factors that played a part here, were: (1) his later acquisition of L2, and/or (2) post-morbid more frequent use of L1. As his language therapy had always been in L2, without much effect in that language prior to inclusion in our study, the improvements we see here after tDCS stimulation are very promising. This is reflected particularly nicely in his picture description scores in L2, which are almost identical at the first three time points of assessment, with then a clear improvement at the fourth assessment, after anodal ctDCS.

Fourth, we found cross-linguistic therapy effects. In both sham and anodal ctDCS, there was cross-linguistic transfer (CLT) to his untreated language with regards to syntactic comprehension. Only after anodal ctDCS, improvements for the repetition task were apparent in the non-treated language (L1), and improvements in untrained picture naming items and picture description were found for both the treated and untreated language. These results align with other intervention studies in bilingual aphasia, reporting transfer from the patients’ trained L2 to their untrained L1 [e.g., (Marangolo et al., 2009; Miertsch et al., 2009; Croft et al., 2011; Kiran et al., 2013; Hameau and Köpke, 2015)]. Importantly, our study is the first to include ctDCS, with indications that this stimulation may further facilitate CLT.

Fifthly, we found transfer of effects to other cognitive abilities, as indicated by performance on a cognitive control task: the Attention Network Test. Here, response time in the congruent condition decreased after anodal ctDCS, but not after sham stimulation. While this does not indicate a specific improvement of inhibition, a main effect of time with a decrease of response times, without a significant interaction effect with condition (congruent/incongruent) may suggest a general improvement in domain-general monitoring abilities (Struys et al., 2019). This adds to a small but growing body of evidence indicating the ability of cerebellar tDCS to affect monitoring (Wynn et al., 2019).

Limitations of this study are that this is a case-study, so results are preliminary. Further, this patient received sham stimulation first, and then anodal ctDCS. In the anodal ctDCS phase, there may have been learning or order effects due to the repeated sessions, lasting from the sham phase affecting test results, for instance in the BNT. Counterbalancing the stimulation order across participants in further studies, or considering practice as a factor in further analyses, may overcome this limitation. In general, the results of this case-study warrant further investigation of tDCS applied to the cerebellum in bilingual patients with aphasia. The cerebellum is generally not a site of damage in post-stroke aphasia, and neither is it in neurodegenerative aphasia, so it may be of interest for neurodegenerative populations as well, such as patients with primary progressive aphasia.

## Conclusion

The results of our case-study, while preliminary, indicate that cerebellar tDCS (ctDCS) may be a useful neuromodulation tool to enhance speech-and language abilities in bilingual patients with aphasia. Picture naming abilities improved after sham and anodal ctDCS but lasted longer after anodal ctDCS. Further, in the anodal ctDCS phase we found generalization across tasks and to the non-treated language. This makes for promising avenues for further studies on ctDCS in aphasia, and on the role of the cerebellum in language.

## Data availability statement

The original contributions presented in this study are included in this article/Supplementary material, further inquiries can be directed to the corresponding author.

## Ethics statement

The studies involving human participants were reviewed and approved by the Commissie Medische Ethiek, Universitair Ziekenhuis Brussel, Brussels, Belgium. The patients/participants provided their written informed consent to participate in this study. Written informed consent was obtained from the individual(s) for the publication of any potentially identifiable images or data included in this article. Written informed consent was obtained from the participant/patient(s) for the publication of this case report.

## Author contributions

SC, SK, and ES were involved in conception and design, analysis, and interpretation of data. SC performed the language evaluation, tDCS treatment, and drafted the manuscript. All authors contributed to manuscript revisions and read and approved the submitted version.
